# The association of dietary inflammatory index with sleep outcomes: A systematic review

**DOI:** 10.34172/hpp.42595

**Published:** 2024-07-29

**Authors:** Mona Golmohammadi, Mehnoosh Samadi, Yahya Salimi, Seyed Mostafa Nachvak, Vahideh Ebrahimzadeh Attari

**Affiliations:** ^1^Student research committee, Department of Nutritional Sciences, School of Nutrition Sciences and Food Technology, Kermanshah University of Medical Sciences, Kermanshah, Iran; ^2^Department of Nutritional Sciences, School of Nutritional Sciences and Food Technology, Kermanshah University of Medical Sciences, Kermanshah, Iran; ^3^Social Development & Health Promotion Research Center, Health Institute, Kermanshah University of Medical Sciences, Kermanshah, Iran; ^4^Department of Clinical Nutrition, Faculty of Nutrition and Food Sciences, Tabriz University of Medical Sciences, Tabriz, Iran

**Keywords:** Dietary patterns, Inflammation, Sleep, Sleep quality

## Abstract

**Background:**

Sleep is a vital physiological process that plays a crucial role in various aspects of human health and well-being. Regarding the important role of diet on the sleep quality, the present study aimed to assess the association of dietary inflammatory index (DII) with the sleep outcomes and also to provide the potential mechanisms of action.

**Methods:**

PubMed, Web of Science and Scopus databases and Google Scholar search engine were systematically searched for relevant studies related to DII and sleep outcomes using appropriate search terms until February 2024.

**Results:**

From the initial systematic search of databases, 197 studies were retrieved. However, only 14 of them met the criteria for evaluation. Out of these, eleven studies indicated a significant correlation between higher DII scores and poor overall sleep quality and/or short/long sleep duration or its subscales. On the contrary, four studies did not find any proof of this association.

**Conclusion:**

This systematic review indicated that following an anti-inflammatory diet could potentially lead to an improvement in the sleep outcomes. Well-designed clinical trials in the future will be necessary to provide a better understanding and quantification of this association.

## Introduction

 Sleep is a state in which a person is immobile and unaware of his or her surroundings.^1^ Adequate sleep leads to recovery of the body and the ability to perform daily activities.^2^ Sleep and wakefulness can be distinguished based on physiological and behavioral differences.^3^

 The International Classification of Sleep Disorders (ICSD-3), established by the American Academy of Sleep Medicine, includes: insomnia, sleep-related breathing disorders, hypersomnolence disorders, circadian rhythm sleep-wake disorders, parasomnias, sleep-related movement disorders, and other sleep disorders^4^ that can affect the quantity and quality of sleep.^5^

 Sleep disorders can be associated with some biochemical variations like: chronic activation of stress hormones,^6,7^ abnormalities in appetite-regulating hormones,^8,9^ increased levels of inflammatory cytokines such as interlukin-6 (IL-6), tumor necrosis factor alpha (TNF-α), IP-10 (an interferon-inducible protein-10 linked to inflammation), and high-sensitivity C-reactive protein (hsCRP),^10^ and changes in gut microbiota.^11^

 Increased stress, somatic pain, decreased quality of life, emotional distress, as well as cognitive deficits and memory impairment are just some of the short-term consequences of sleep disorders. Moreover, chronic sleep disorders can increase the risk of hypertension, dyslipidemia, cardiovascular disease, obesity, metabolic syndrome, diabetes and cancer.^12^

 Many factors affect the sleep quality, and diet is one of the most important factors. The relationship between diet and sleep is a bidirectional effect. Sleep deprivation increases hunger and appetite via the homeostatic system^13^ and activates the hedonic system (reward system) in the brain, leading to the obesity.^14^ On the other hand, diet and nutrients also affect the sleep quality. Tryptophan (TRP) is the precursor of serotonin and melatonin synthesis, both of which are associated with sleep and wakefulness.^15^ It was shown that, TRP rich foods like meats, dairy, fruits, and seeds may have positive effects on the mood and sleep quality.^16^

 The presence of carbohydrates and proteins are also necessary for melatonin and serotonin synthesis from TRP.^17^ Protein rich foods are good source of large neutral amino acid (LNAA) along with TRP that compete each other to cross the blood-brain barrier (BBB)^18^ and a high TRP /LNAA ratio is important in the serotonin and melatonin synthesis.^19^ Although animal foods contain more protein and TRP, the TRP /LNAA ratio is higher in plant proteins.^20^ Moreover, some dietary bioactive peptides like α-s1-casein hydrolysate interact with GABAergic or serotonergic neurons which affect the sleep state.^21^

 Moreover, there are several dietary patterns that can impact sleep, including a Mediterranean diet, which has been shown to be associated with better sleep quality and/or duration.^22^ The Mediterranean diet is an antioxidants rich diet that includes fruits, vegetables, whole grains, legumes, nuts, and healthy fats.^23^ In contrast, the Western diet, which is high in animal proteins, saturated fats, and foods rich in refined sugars, leads to poor sleep quality.^24^ Nowadays, the dietary inflammatory index (DII) is used as an effective tool to represent the inflammatory potential of foods. High DII levels are associated with pro-inflammatory diets, while low DII levels are associated with anti-inflammatory diets.^25^ The association between sleep and the inflammatory potential of diet is one of the important research areas that plays an important role in human health. Some studies have investigated the association between DII and sleep status, but to our knowledge there is not yet conclusive results.^26-29^ Therefore, the present study aimed to systematically review the association of DII and sleep status and also to provide the potential mechanisms of action.

## Methods

###  The search strategy

 The present systematic review was conducted in accordance with the PRISMA-P (Preferred Reporting Items for Systematic Reviews and Meta-Analyses Protocols) 2015 Statement.^30^ We conducted a comprehensive search of PubMed/Medline, Web of Science and Scopus databases and Google Scholar search engine published through February 2024 by combining the related key terms of DII and sleep. The search was conducted using both MeSH terms and free keywords. The complete search strategy was shown in Table S1 (Supplementary file 1).

###  The screening of studies

 Two reviewers (MG and SMN) independently reviewed articles according to the inclusion criteria and excluded the irrelevant articles by reviewing the title and abstract of the articles. Two researchers (MG and SMN) read the full text of the remaining articles to verify their eligibility for the study and to extract data. The additional relevant studies were also identified using the articles’ reference list. The third author clarified all discrepancies between the two authors (YS). All retrieved studies saved in an EndNote library and duplicate references were deleted.

###  Inclusion and exclusion criteria

 All relevant studies, regardless of their publication date, and all observational or experimental research which were written in English, were included in the study. While the animal studies, in vitro models, conference abstracts, review studies, protocols, and those lacking enough data on the association between DII and sleep status were excluded.

###  Data extraction and quality assessment

 A standard extraction form was used to collect data based on author’s name, country of origin, type of research, population/sample size, mean age of participants, type and duration of intervention, assessment tools for sleep/DII/food intake, confounding variables, and main outcomes.

 The adapted Newcastle-Ottawa Scale (NOS) and the Jadad checklists were used to assess the quality of articles in cross-sectional/case-control studies and experimental studies, respectively. Tables S2 and S3 present the quality assessment results of cross-sectional and case-control studies, while Table S4 presents the results of experimental studies. The NOS consists of the domains of selection, comparability and result, which can be rated with values between 0 and 9. According to the results of the NOS checklist, scores of seven or higher were classified as minimal risk of bias, scores between four and six were classified as high risk of bias, and scores below four were classified as very high risk of bias. The Jadad checklist comprises five criteria, each of which is rated as “yes” or “no”. The total score for the checklist can range from 0 to 5. Based on the Jadad checklist, scores of three or higher were interpreted as having superior quality.

## Results

###  Selection of studies and characteristics of included studies

**Figure 1:**
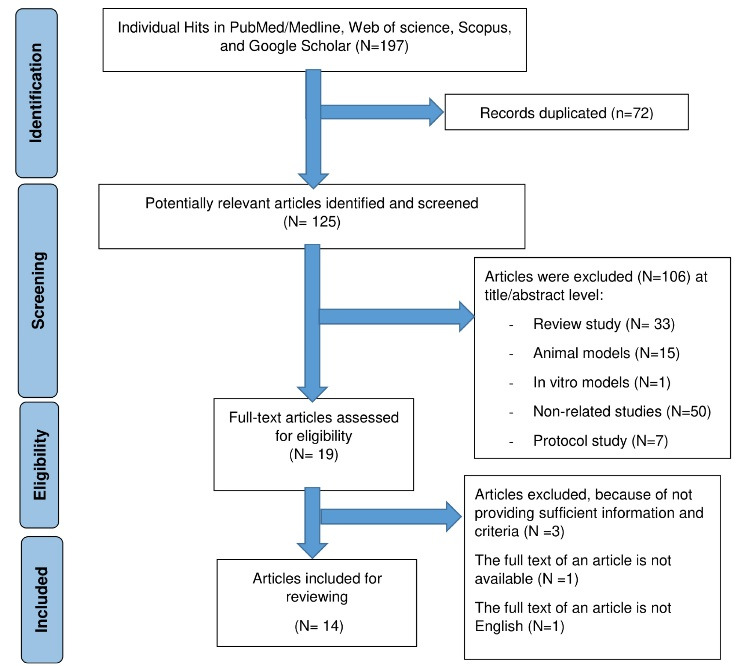
Preferred Reporting Items for Systematic Reviews and Meta-Analyses (PRISMA) flow diagram with information about selection process of studies included in the review

 The search strategy identified 197 potentially relevant articles, of which 72 were duplicates and excluded, leaving 125 articles. After further screening, 106 were removed at the title/abstract level. Finally, 14 studies were included in the final review (see Figure 1).

 The studies included in this review had the following participants: adult healthy population (n = 7),^28,31-36^ students (n = 2),^27,37^ obese or overweight women (n = 2),^26,38^ fibromyalgia syndrome (n = 1),^39^ obstructive sleep apnea (OSA) (n = 1),^29^ ulcerative colitis (UC) (n = 1).^40^Table 1 provides an overview of the details of each study.

 Most subjects in the studies were adults over 18 years of age. The gender of the subjects was male and female in all studies, except in 4 studies in which the subjects were female.^26,27,38,39^ Most studies were cross-sectional (n = 11),^26-29,31-34,36,37,40^ with the exception of three studies, which were a self-selection^35^ study and a randomized controlled trial (RCT)^38^ lasting 12 and 16 weeks, and a case-control study.^39^ The studies were conducted in different countries, including 6 studies in the USA,^33-36,38,40^ 3 studies in Iran,^26,27,32^ 1 study in China,^31^ 1 study in Spain,^39^ 1 study in Brazil,^29^ 1 study in the UAE ^37^ and 1 study in Italy^28^ (see Table 1).

 There was some heterogeneity between articles in terms of assessment of sleep status and DII. Different subjective and objective methods were used to examine people’s sleep status.For subjective assessment, questionnaires such as the Pittsburgh Sleep Quality Index (PSQI),^26-28,31,32,34,36,37,39^ and the Health Patient Reported Outcome Measurement Information System (PROMIS)^40^ were used. In addition, instruments such as the Micro Motion Logger Sleep Watch,^33^ Polysomnography (PSG),^29^ and SenseWear armband^35,36,38^ were used for the objective assessments. The sleep status was assessed subjectively in 9 studies,^26-28,31,32,34,37,39,40^ objectively in 2 studies,^35,38^ and both subjectively and objectively in three studies.^29,33,36^ In addition, DII was assessed using short dietary assessment instrument,^40^ food frequency questionnaires (FFQ)^26-29,32,33,37^ and/or 3-day,^35,36^ 2-day,^31,38^ or one-day 24-hour^34,39^ food recall questionnaires.

**Table 1:** Summary of included studies

**Author, Country**	**Type of research **	**Population/Sample size**	**Age (y)**	**Interventional diet**	**Control diet**	**Length of intervention (wk)**	**Sleep status assessment tools**	**DII assessment method**	**Food intake assessment tools**	**Confounding variables**	**Main Outcomes**
DuBois et al, USA^40^	Cross-sectional	Patients with UC male/female n = 2052	43.8 ± 14.4	-	-	-	PROMIS	45 food parameters	Short dietary assessment instrument (26 items)	Age, sex, BMI, race, education, smoking status, medication class, disease duration, and physical activity	Sleep disturbance was positively associated with increased E-DII score (*P* = 0.003)
Farrell et al, USA^36^	Longitudinal	Adult population male/female n = 427	27.6 ± 3.8	-	-	-	1. PSQI 2. BodyMedia’s SenseWear Armband	44 food parameters	24-hour dietary recall (three days)	BMI, WHR, blood pressure, blood composition, gender, education, income, employment status, marital status, children, race, age, physical activity, and sedentary hours	WASO (*P* = 0.02), bedtime (*P* < 0.01), and waketime (*P* < 0.01) were positively associated with increased DII score. Each unit increase in change in DII score was positively associated with increased WASO (*P* = 0.01), decreased sleep efficiency (*P* = 0.05), later bedtime (*P* = 0.04), and later waketime (*P* = 0.04).
Wang et al, China^31^	Cross-sectional	US adult population male/female n = 5594	≥ 30	-	-	-	PSQI	26 food parameters	24-hour dietary recall (two days)	Age, gender, race, physical activity, smoking status, BMI, waist circumference, and energy intake	In the subjects with poor sleep quality, PSQI was positively associated with increased DII score (*P* < 0.001)
Bavi Behbahani et al, Iran^32^	Cross-sectional	Employees male/female n = 211	38.8 ± 11.3	-	-	-	PSQI	45 food parameters	FFQ (147 items)	Age, BMI, and energy intake	Sleep duration was negatively associated with increased DII score (*P* < 0.001).
Wirth et al, USA^33^	Cross-sectional longitudinal	Police officers male/ female n = 464	41.5 ± 6.7	-	-	-	1. PSQI 2. Micro Motion Logger Sleep Watch	29 food parameters	FFQ (144 items)	PSQI: years of employment as a police officer, center for epidemiologic studies depression scale, beck anxiety inventory, the impact of events WASO: tabacco use, BMI, systolic blood pressure, years of employment as a police officer, waist circumference, average number of alcoholic drinks per week, average day shift hours per week	PSQI was negatively associated with increased DII score (*P* = 0.01) WASO was positively associated with increased DII score (*P =*0.02). An increase of 1 unit in E-DII scores over time was associated with an increase in WASO scores (*P* = 0.07) and an improvement in PSQI scores (*P* < 0.01).
Masaad et al, UAE^37^	Cross-sectional	College students male/female n = 379	18-30	-	-	-	PSQI	38 food parameters	FFQ (94 items)	-	There was no significant association between DII score and most parameters of sleep quality, except for day dysfunction (*P* = 0.01).
Kase et al, USA^34^	Cross-sectional	US adult population male/female n = 23867	47.2 ± 0.3 (SE)	-	-	-	By the question	45 food parameters	24-hour diet recall (single day)	Age, sex, ethnicity, education level, marital status, BMI, and chronic disease	The DII score was significantly higher among participants who had either short or long sleep duration (*P* < 0.001).
Pourteymour Fard Tabrizi et al, Iran^26^	Cross-sectional	Reproductive-aged women with obesity or overweight female n = 278	31.4 ± 10.9	-	-	-	PSQI	24 food parameters	FFQ (168 items)	-	There was no association between PSQI and DII score (*P* = 0.76).
Bazyar et al, Iran^27^	Cross-sectional	Students female n = 249	23.9 ± 3.8	-	-	-	PSQI	30 food parameters	FFQ (147 items)	Age, energy intake, physical activity, and education	PSQI was positively associated with increased DII score (*P* = 0.02).
Godos et al, Italy^28^	Cross-sectional	Adults male/ female n = 1936	≥ 30	-	-	-	PSQI	33 food parameters	FFQ (100 items)	-	A lower percentage of participants with higher sleep quality was found in the higher quartiles of DII (*P* = 0.03).
Lopes et al, Brazil^29^	Cross-sectional	Patients with OSA male/ female n = 296	18-60	-	-	-	1. PSQI 2. PSG	27 food parameters	FFQ (27 items)	PSQI: BMI, waist and neck circumferences, physical activity PSG: diastolic blood pressure, marital status, smoking habit, waist and neck circumferences, carbohydrates intake, physical activity, protein intake, systolic blood pressure, sex, BMI, apnea-hypopnea index, age, household income, education, fat intake, work status, napping, alcohol consumption	There was no association between PSQI and DII score (*P* > 0.05). There was no association between PSG and DII score (*P* = 0.16).
Correa-Rodríguez et al, Spain^39^	Case-Control	Patients with fibromyalgia syndrome (case) female n = 95 menopause woman (control) n = 98	Case: 55.8 ± 8.0 Control: 55.1 ± 10.3	-	-	-	PSQI	23 food parameters	24-hour diet recall (single day)	Age, menopause status and total energy	There was no association between PSQI and DII score in either groups. Case: *P* = 0.18 Control: *P* = 0.95
Wirth et al, USA^38^	RCT	Pregnant women who were overweight or obese before pregnancy (n = 207)	29.8 ± 5.0	Healthy diet + active living + weight monitoring	Receive standard prenatal care from their provider, which may have included nutrition and physical activity-related information and service	16 weeks	BodyMedia’s SenseWear Armband	27 food parameters	24-hour diet recall (two days)	Vitamin usage, social support, steps per day, sedentary time per day, ever-smoke status, parity, race, insurance of mother, physical activity social support from family and friends, dietary social support from family and friends, children in household 5-17 years, perceived stress, fast food consumption, income, employment status, and moderate-to-vigorous physical activity per day	A positive association was observed between a higher DII score and a longer sleep latency (*P* < 0.01). Among European Americans, a positive association was observed between a higher DII score and increased WASO (*P* = 0.02)
Wirth et al, USA^35^	Self-selection trial	US adult population male/female Intervention(n = 61) Control(n = 34)	Intervention: 51.1 ± 11.0 Control: 39.2 ± 14.0	Anti-inflammatory plant-based foods + physical activity + Stress management	Cancer prevention educational	12 weeks	Validated SenseWear Armband	43 food parameters	24-h dietary recalls (three days)	Age, years exposed to shiftwork throughout lifetime, and perceived health	Those with anti-inflammatory changes experienced a decrease in WASO (*P* < 0.01) and an improvement in sleep efficiency (*P* = 0.04).

Abbreviations: BMI, body mass index; DII, dietary inflammatory index; FFQ, food frequency questionnaires; OSA, obstructive sleep apnea; PROMIS, health patient reported outcome measurement information system; PSG, polysomnography; PSQI, Pittsburgh sleep quality index; RCT, randomized control trial; UC, ulcerative colitis; WASO, wake after sleep onset; WHR, waist to hip ratio.

###  Quality of the articles

 According to the NOS checklist, all cross-sectional/case-control studies had a minimal risk of bias. The Jadad checklist also found that all intervention studies were of inferior quality. Tables S2, S3, and S4show the quality results of the studies.

###  Association between the DII and sleep status

 Nine of eleven cross-sectional studies revealed a significant correlation between higher DII scores and overall poor sleep quality and/or changes in sleep duration or its subscales.^27,28,31-34,36,37,40^ However, two cross-sectional studies showed no association between DII and sleep status. These two studies were conducted in women with overweight or obesity,^26^ and patients with OSA.^29^ There was also no association between DII and sleep quality using the PSQI questionnaire in the case-control study.^39^ It is important to note that the association between DII and sleep quality may be influenced by the underlying illness status of the participant in the studies, and this should be carefully considered in the interpretation of the findings.

 In addition, the results of the included self-selection study and RCT showed that taking an anti-inflammatory diet for 12 and 16 weeks significantly improved the sleep status of patients.^35,38^

**Figure 2:**
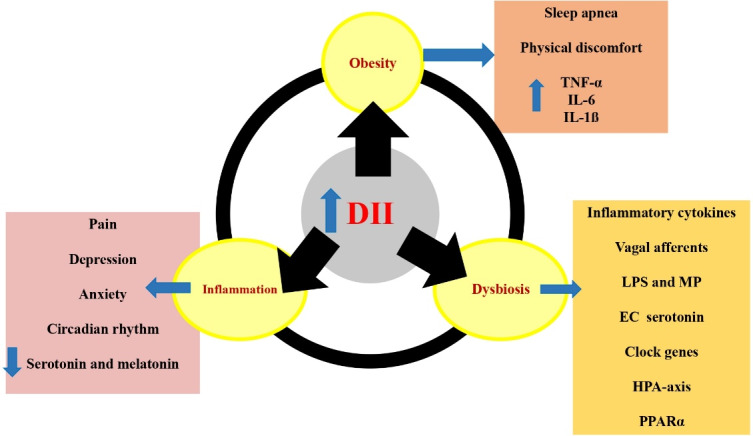
The potential mechanisms behind the effects of pro-inflammatory diets on sleep outcomes. Pro-inflammatory diets are associated with increased obesity, inflammation and dysbiosis, which causes sleep apnea, increase of inflammatory cytokines, anxiety, depression, disruption of circadian rhythms, reduction of serotonin and melatonin secretion, stimulation of vagal afferents, translocation of LPS and MP, activation of HPA axis and PPARα. EC: enterochromaffin, DII: dietary inflammatory index, HPA: hypothalamic-pituitary-adrenal, IL: interleukin, LPS: lipopolysaccharide, MP: Muramyl peptide, PPARα: Peroxisome proliferator-activated receptor alpha, TNF-α: tumor necrosis factor alpha

 Possible mechanisms for the effects of a pro-inflammatory diet on sleep outcomes are summarized in Figure 2 and discussed in the following 3 sections.

## Discussion

 To our knowledge, the present systematic review is the first comprehensive investigation to evaluate the association between DII and sleep status. The majority of studies suggested that there is a negative association between DII scores and sleep outcomes, as shown in this systematic review.

 Most of the studies included in this review were cross-sectional studies, and most of these studies showed an association between the DII and sleep outcomes or its subscales, with the exception of two studies, one of which was Lopes and colleagues’ study,^29^ in which participants suffered from OSA, which likely has an indirect effect between dietary habits and OSA, possibly mediated by overweight and obesity. In this study, the 27-item FFQ was used, and the accurate assessment of the DII with 27 items may not be accurate enough. The next study was the Tabrizi study,^26^ whose study population consisted of overweight and obese people, and this overweight and obesity itself may cause sleep disturbances. It is worth noting that these studies do not show a causal relationship due to their cross-sectional nature. In Ferrell’s study,^36^ which was a longitudinal study, a one-unit increase in DII was associated with an increase in wake after sleep onset (WASO), a decrease in sleep efficiency, later bedtime and later wakeup. Considering the longitudinal nature of this study and the use of the SenseWear armband to measure sleep quality, the results of this study may confirm the findings that showed the negative relationship between DII and sleep quality. In addition, in Wirth’s study,^33^ a one-unit increase in DII over time was associated with an increase in WASO, but in the case of PSQI, the results showed something different, namely improvement in PSQI over time. The participants in this study were police officers who worked shifts. They might leave their jobs over time or move to a different shift, which could bias the results, as those remaining are likely to be genetically better able to deal with the adverse effects of shift work. On the other hand, the PSQI is a subjective instrument that can influence people’s self-assessment because they want to better represent their health. For example, it was reported that subjects’ total sleep time was longer with self-report than with actigraphy.^41^ In this systematic review, two intervention studies were conducted that showed causal relationships.^35,38^ In addition, these studies used the SenseWear armband was used to assess sleep quality, and the results confirmed the findings of most cross-sectional studies. Therefore, it can be concluded that an anti-inflammatory diet can be used as a strategy to improve sleep.

 The DII is a useful tool that determines the degree of inflammation in the diet based on its content of pro-inflammatory and anti-inflammatory nutrients.^25^ It should be noted that a pro-inflammatory diet with high DII score has a lower amount of some nutrients such as magnesium and folic acid, as this diet is low in fruits and vegetables.^42^ On the other hand, these nutrients can affect a person’s sleep quality. Magnesium is an important mineral that has a positive effect on nerve function and sleep quality.^43,44^ Magnesium may regulate melatonin production by increasing the activity of N-acetyltransferase (NAT), which plays a critical role in controlling the sleep-wake cycle.^45^ Furthermore, magnesium has been identified as an N-methyl-D-aspartic acid (NMDA) antagonist and GABA agonist that may influence sleep behavior.^46,47^ Vitamin B9 (also known as folic acid) and vitamin B12 also play an important role in the production of neurotransmitters, including serotonin, which is involved in regulating mood and sleep, and melatonin, which regulates the sleep-wake cycle.^16,48^

 Moreover, a pro-inflammatory high glycemic index (GI) diet with high amount of refined carbohydrates, can cause compensatory hyper-insulinemia. This can lead to the release of autonomic counter regulatory hormones such as growth hormone, epinephrine, glucagon, and cortisol. These hormones may contribute to the development of insomnia.^49^ In addition, a diet high in GI has been shown to trigger inflammatory immune responses and alter the gut microbiota, which could affect sleep quality.^49^

 One of the characteristics of diets with a high inflammatory index is the high consumption of fats. Increased fat intake decreases the need for lipid and cholesterol synthesis. This may delay phosphorylation of eukaryotic initiation factor 2α (elF2α), as a sleep signaling agent.^50,51^

 Our results are also consistent with previous review studies that assessed the association of dietary patterns with sleep quality. It was reported that following a healthy dietary patterns such as the Mediterranean diet is associated with sufficient sleep and improved sleep quality through effect on different biological processes like the inflammation, cell signaling, metabolism, and oxidative stress.^22^ Another review study, which included observational studies, showed that adherence to the Mediterranean dietary pattern is associated with better sleep quality, adequate sleep duration and other sleep parameters, such as: less sleepiness during the day.^52^ The Mediterranean diet is one of the diets with a low inflammatory index, which, due to its high content of antioxidants and polyphenols, affects brain health in several ways, including reducing the production of inflammatory cytokines and protecting neurons by activating the nuclear factor erythroid 2-related factor 2 (Nrf2) and stimulating receptors of the sirtuin family.^49,53^ In addition, this dietary pattern is rich in omega-3 fatty acids and monounsaturated fatty acids, and the amount of saturated and trans fatty acids is low, which reduces inflammation in the body by decreasing Toll-like receptors (TLRs) on the surface of microglia in the brain.^52,54^ In addition, a systematic review study has shown that a healthy diet is associated with better sleep quality.^55^ These healthy eating patterns are characterized by a higher intake of fruits, vegetables, seafood, legumes and whole grains and a lower intake of processed and sugary foods. The characteristics of these dietary patterns are their low-inflammatory properties. These results were also confirmed in a narrative review study.^56^ On the other hand, evidence indicates that consumption of a Western diet characterized by high intake of processed foods, refined sugars, and saturated fats can disrupt the body’s natural rhythm of cortisol release and is associated with poor sleep quality and increased risk of sleep disturbance.^57,58^

###  DII and obesity

 Studies have found that a pro-inflammatory diet, as indicated by high DII scores, is positively associated with obesity^59,60^ and other related health problems such as metabolic syndrome,^61^ insulin resistance, and type 2 diabetes.^62^ A prospective study conducted on a population over a 10-year period found that individuals who had higher levels of fibrinogen, CRP and WBC at baseline were more likely to experience significant annual weight gain compared to those with lower levels of these inflammatory markers.^63^ Obesity, especially morbid obesity with the body mass index (BMI) more than 35 can increase the risk of developing sleep apnea. Sleep apnea leads to frequent nighttime awakenings and excessive daytime sleepiness.^64^ This sleep impairment can induce hyperphagia and increase weigh in turn as a defective cycle. Moreover, some of the consequences of excessive body weight like joint pain or gastro-esophageal reflux disease can also interfere with sleep.^65-68^ Visceral adipose tissue also increases the production of cytokines such as TNF-α, IL-6, and IL-1ß which have the potential to disrupt sleep.^68^

###  DII and inflammation

 The DII takes into account the anti-inflammatory and pro-inflammatory effects of various foods and nutrients and assigns a score to each food item based on its overall inflammatory potential. Foods high in sugar, saturated fat, and processed foods tend to have higher DII scores, while foods rich in antioxidants, fiber, and healthy fats tend to have lower scores.^25^ Foods with higher DII levels are associated with increased levels of TNF-α, IL-6, and IL-1ß in the body.^25^ They can significantly affect the sleep quality and leads to malaise, depression, and anxiety.^69^ Pro-inflammatory diet causes expression of inflammatory mediators by binding to the TLR4 receptor through two pathways, nuclear factor-κB (NF-κB) and the c-Jun N-terminal kinase (JNK) pathway. Peroxisome proliferator-activated receptor γ (PPARγ) can inhibit the NF-κB pathway, and anti-inflammatory foods enhance its effect by binding to PPARγ. Anti-inflammatory diet reduces inflammation by activating Nrf2. Nrf2 is a transcription factor that is activated by cleavage from Kelch-like ECH-associated protein 1 (Keap1) and after entering the nucleus and binding to Maf proteins (musculoaponeurotic fibrosarcoma) and to the antioxidant response element for expression of phase 2 antioxidant enzymes.^70^ Moreover, immune and inflammatory mediators can directly affect genes regulating circadian rhythm activity including CLOCK (Circadian Locomotor Output Cycles Kaput) and PER (Period).^71^ In addition, inflammation affects the secretion of hormones like serotonin and melatonin, resulting in both depression and disruption of sleep patterns.^72-74^

###  DII and microbiota modulation

 Diet is considered a modifiable factor affecting the gut microbiome. Dysbiosis, which is an imbalance in the microbial environment, has been linked to a number of diseases including Alzheimer’s, Parkinson’s, multiple sclerosis, and sleep disorders.^75,76^ High DII can lead to a decrease in microbial diversity, an increase in inflammation, changes in the composition of the gut microbiota, and dysbiosis.^77,78^ The gut microbiome has the ability to influence the sleep through translocation of somnogenic lipopolysaccharide (LPS) and muramyl peptide (MP), stimulating vagal afferents through the enteric LPS response, controlling enterochromaffin serotonin production, and regulating inflammatory cytokines ^79^. Moreover, metabolites produced by the gut microbiota influence the expression of clock genes in central nervous system and hepatic regions. Dysbiosis can lead to changes in sleep patterns, such as fragmented or shortened sleep, due to activation of the hypothalamic-pituitary-adrenal (HPA) axis ^79^. Moreover, the absence of gut microbiota leads to the activation of peroxisome proliferator-activated receptor alpha (PPARα) and impairs the function of Bmal1 and Cry1 genes which, regulate the body’s internal clock.^80^

## Strength of the study

 To our knowledge the present systematic review is the first comprehensive investigation to examine the association between DII as a dietary quality index and sleep status. While, previous review articles studied the association of dietary patterns with sleep quality.

## Limitations of the study

 The results presented in this systematic review should be considered with some limitations: First, most studies used a cross-sectional design, making it difficult to examine the causal relationship between variables. Second, studies used different methods to assess both DII and sleep status, resulting in the lack of consistency in measurement tools. Third, most studies did not use objective instruments such as PSG and actigraphy to examine sleep state. Forth, the accuracy of self-reports assessment of food intake and sleep quality is limited as there may be a recall bias.

## Conclusion

 In conclusion, this systematic review indicated that an anti-inflammatory diet can improve sleep quality. However, more rigorous clinical trials are needed to better understand the extent of this relationship and to draw more definitive conclusions. Moreover, an important aspect is the generalizability of the results. As the populations studied in this systematic review include a wide range of individuals, including both healthy and sick individuals, who come from different age groups and countries with different racial, genetic and socioeconomic backgrounds. These inherent differences should be carefully considered to ensure accurate interpretation and application of the results in different populations.

## Clinical and research implications

 To discuss the clinical and research implications of these findings, it is essential to consider practical strategies that may benefit people with sleep problems. One possible approach is the screening of individuals with sleep problems and a comprehensive review of their diet, particularly in relation to the DII, by trained experts. In addition, nutrition counseling of patients with sleep disorders could help people improve their lifestyle and reduce the burden of disease.

## Future clinical and research directions

 The following directions are suggested for future research:

1. Future well designed clinical trials with larger sample size and longer duration are needed to demonstrate a causal relationship between DII and sleep.
2. More observational studies in Asian countries are needed to investigate the association between DII and sleep outcomes among them.
3. Objective instruments such as actigraphy and polysomnography should be used in future studies.

## Acknowledgments

 We wish to express our appreciation to the Research Vice-Chancellor of Kermanshah and Tabriz Universities of Medical Sciences.

## Competing Interests

 The authors declare no competing interests.

## Declaration of Generative AI

 During the preparation of this paper the authors used Perplexity AI to write a discussion. After using this tool, the authors reviewed and edited the content as needed and take full responsibility for the content of the publication.

## Ethical Approval

 This study is part of the Ph.D thesis approved by the Research Ethics Board of Kermanshah University of Medical Sciences (IR.KUMS.REC.1402.170).

## Supplementary Files


Supplementary file 1 contains Table S1-S4.
