# Supplementary material for: The association of dietary inflammatory index with sleep outcomes: A systematic review
Source: Health Promot Perspect. 2024 Jul 29;14(2):136–47. doi: 10.34172/hpp.42595 (PMC11403336; doi:10.34172/hpp.42595)
Supplement: Supplementary file 1 — contains Table S1-S4. [file hpp-14-136-s001.pdf]

Golmohammadi et al, **Health Promotion Perspectives,** 2024, 14(2), 136-S1
doi: 10.34172/hpp.42595
https://hpp.tbzmed.ac.ir

Supplementary Material

Supplementary Tables

Supplemental Table S1. Search strings by databases

Database	Search string
PubMed/Medline	("dietary inflammatory index"[Title/Abstract] OR "dietary inflammatory score"[Title/Abstract] OR "dietary inflammation potential"[Title/Abstract] OR "anti inflammatory diet"[Title/Abstract] OR "DII"[Title/Abstract] OR ((("potential"[All Fields] OR "potential s"[All Fields] OR "potentialities"[All Fields] OR "potentiality"[All Fields] OR "potentially"[All Fields] OR "potentials"[All Fields] OR "potentiate"[All Fields] OR "potentiated"[All Fields] OR "potentiates"[All Fields] OR "potentiating"[All Fields] OR "potentiation"[All Fields] OR "potentiations"[All Fields] OR "potentiative"[All Fields] OR "potentiator"[All Fields] OR "potentiators"[All Fields]) AND ("inflammatories"[All Fields] OR "inflammatory"[All Fields])) AND "intake"[Title/Abstract]) OR "inflammatory diet"[Title/Abstract] OR "pro inflammatory diet"[Title/Abstract] OR "inflammatory potential of diet"[Title/Abstract]) AND ("sleep"[MeSH Terms] OR "long sleep duration"[Title/Abstract] OR "sleep duration"[Title/Abstract] OR "night time sleep duration"[Title/Abstract] OR "short sleep"[Title/Abstract] OR "short sleep duration"[Title/Abstract] OR "sleep quality"[Title/Abstract] OR "insomnia"[Title/Abstract] OR "sleep disorders"[Title/Abstract] OR "short term sleep restriction"[Title/Abstract] OR "drowsiness"[Title/Abstract] OR "hypersomnia"[Title/Abstract] OR "sleep disturbance"[Title/Abstract] OR "daytime sleepiness"[Title/Abstract] OR "sleep time"[Title/Abstract] OR "sleep"[Title/Abstract] OR "short sleepers"[Title/Abstract] OR "sleep deprivation"[Title/Abstract] OR "long sleepers"[Title/Abstract] OR "sleep disorders"[Title/Abstract] OR "sleep time"[Title/Abstract] OR "nap"[Title/Abstract])
Web of Science	TS=("dietary inflammatory index" OR "dietary inflammatory score" OR "dietary inflammation potential" OR "anti-inflammatory diet" OR "DII" OR "potential inflammatory intake" OR "inflammatory diet" OR "pro-inflammatory diet" OR "inflammatory potential of diet") AND TS=("long sleep duration" OR "sleep duration" OR "night time sleep duration" OR "short sleep" OR "short sleep duration" OR "sleep quality" OR insomnia OR "sleep disorders" OR "Short-term sleep restriction" OR drowsiness OR hypersomnia OR "sleep disturbance" OR "daytime sleepiness" OR "sleep time" OR sleep OR "short sleepers" OR "sleep deprivation" OR "long sleepers" OR "sleep disorders" OR "sleep time" OR nap)
Scopus	TITLE-ABS-KEY("dietary inflammatory index") OR TITLE-ABS-KEY("dietary inflammatory score") OR TITLE-ABS-KEY("dietary inflammation potential") OR TITLE-ABS-KEY("anti-inflammatory diet") OR TITLE-ABS-KEY(DII) OR TITLE-ABS-KEY("potential inflammatory intake") OR TITLE-ABS-KEY("inflammatory diet") OR TITLE-ABS-KEY("pro-inflammatory diet") OR TITLE-ABS-KEY("inflammatory potential of diet") AND TITLE-ABS-KEY("long sleep duration") OR TITLE-ABS-KEY("sleep duration") OR TITLE-ABS-KEY("night time sleep duration") OR TITLE-ABS-KEY("short sleep") OR TITLE-ABS-KEY("short sleep duration") OR TITLE-ABS-

	KEY("sleep quality") OR TITLE-ABS-KEY(insomnia) OR TITLE-ABS-KEY("sleep disorders") OR TITLE-ABS-KEY("Short-term sleep restriction") OR TITLE-ABS-KEY(drowsiness) OR TITLE-ABS-KEY(hypersomnia) OR TITLE-ABS-KEY("sleep disturbance") OR TITLE-ABS-KEY("daytime sleepiness") OR TITLE-ABS-KEY("sleep time") OR TITLE-ABS-KEY(sleep) OR TITLE-ABS-KEY("short sleepers") OR TITLE-ABS-KEY("sleep deprivation") OR TITLE-ABS-KEY("long sleepers") OR TITLE-ABS-KEY("sleep disorders") OR TITLE-ABS-KEY("sleep time") OR TITLE-ABS-KEY(nap)

Supplemental Table S2. The adapted version of the Newcastle–Ottawa Scale (NOS) Checklist for Cross-Sectional/Longitudinal studies

**Author, publication year**	**Study design**	**Selection**				**Comparability**	**Outcome**			
		**Representativeness of the sample**	**Sample size**	**Nonrespondents**	**Ascertainment of exposure**	**Based on design and analysis**	**Assessment of outcome**	**Statistical test**	**Score**	**Interpretation**
DuBois et al., 2024 [40]	Cross-sectional	+	+		++	++	+	+	8	**Low risk of bias**
Farrell et al., 2023 [36]	Longitudinal	+	+		++	++	++	+	9	**Low risk of bias**
Wang et al., 2022 [31]	Cross-sectional	+	+		++	++	+	+	8	**Low risk of bias**
Behbahani et al., 2022 [32]	Cross-sectional	+	+		++	++	+	+	8	**Low risk of bias**
Wirth et al., 2022 [33]	Cross-sectional	+	+		++	++	++	+	9	**Low risk of bias**

Masaad et al., 2021 [37]	Cross-sectional	+	+		++	+	+	+	7	**Low risk of bias**
Kase et al., 2021 [34]	Cross-sectional	+	+	+	+	++	+	+	8	**Low risk of bias**
Tabrizi et al., 2021 [26]	Cross-sectional	+	+		++	+	+	+	7	**Low risk of bias**
Bazyar et al., 2021 [27]	Cross-sectional	+	+	+	++	++	+	+	9	**Low risk of bias**
Godos et al., 2019 [28]	Cross-sectional	+	+		++	++	+	+	8	**Low risk of bias**
Lopes et al., 2019 [29]	Cross-sectional		+		++	+	++	+	7	**Low risk of bias**

Supplemental Table S3. The adapted version of the Newcastle–Ottawa Scale (NOS) Checklist for Case-Control studies

**Author, publication year**	**Study design**	**Selection**				**Comparability**	**Outcome**				
		**The definition of cases is adequate**	**Representativeness of the cases**	**Selection of controls**	**Definition of controls**	**Based on design and analysis**	**Ascertainment of exposure**	**Same method of ascertainment for cases and controls**	**Non-response rate**	**Score**	**Interpretation**

Correa-Rodríguez et al., 2020 [39]	Case-Control	+	+	+	+	++		+		7	**Low risk of bias**

Supplemental Table S4. The Jadad Checklist for Randomized Controlled Trials

**Author, publication year**	**Study design**	**Was the study described as randomized (this includes words such as randomly, random, and randomization)?**	**Was the method used to generate the sequence of randomization described and appropriate (table of random numbers, computer-generated, etc)?**	**Was the study described as double blind?**	**Was the method of double blinding described and appropriate (identical placebo, active placebo, dummy, etc)?**	**Was there a description of withdrawals and dropouts?**	**Score**	**Interpretation**
Wirth et al., 2022 [38]	RCT	1	0	0	0	1	2	inferior quality
Wirth et al., 2020 [35]	Self-selection trial	0	0	0	0	0	0	inferior quality
